# Correction: Effects of electrodeposition time on a manganese dioxide supercapacitor

**DOI:** 10.1039/d0ra90053b

**Published:** 2020-05-11

**Authors:** Xiaoli Dai, Ming Zhang, Jitao Li, Dingyu Yang

**Affiliations:** College of Optoelectronic Technology, Chengdu University of Information Technology Chengdu 610225 China yangdingyu@cuit.edu.cn; School of Precision Instruments and Optoelectronics Engineering, Tianjin University Tianjin 300072 China jtlee@tju.edu.cn

## Abstract

Correction for ‘Effects of electrodeposition time on a manganese dioxide supercapacitor’ by Xiaoli Dai *et al.*, *RSC Adv.*, 2020, **10**, 15860–15869, DOI: 10.1039/D0RA01681K.

The authors regret that the email addresses of two authors (Dingyu Yang and Jitao Li) were shown incorrectly in the original article. The correct email addresses are as shown above.

The Royal Society of Chemistry apologises for these errors and any consequent inconvenience to authors and readers.

## Supplementary Material

